# Clinical Relevance of Lipoprotein(a) in Young Acute Myocardial Infarction: STEMI vs. NSTEMI

**DOI:** 10.3390/biomedicines13112662

**Published:** 2025-10-30

**Authors:** Silvana Isabella Cureraru, Alexandru Mugurel Belu, Eugen Nicolae Țieranu, Ionuț Cezar Buciu, Mina Teodora Piorescu, Ionuț Donoiu, Maria Iovănescu, Georgică Costinel Târtea, Cristian Militaru, Petre Alexandru Cojocaru, Octavian Istratoaie

**Affiliations:** 1 Doctoral School, University of Medicine and Pharmacy of Craiova, 200349 Craiova, Romania; isabellacureraru@yahoo.ro; 2 Department of Cardiology, University of Medicine and Pharmacy of Craiova, 200349 Craiova, Romania; ionut.donoiu@umfcv.ro (I.D.); maria.iovanescu@umfcv.ro (M.I.); george.tartea@gmail.com (G.C.T.); cojo.alexandru92@gmail.com (P.A.C.); droctavist@yahoo.com (O.I.); 3 Department of Cardiology, Emergency County Hospital, 299642 Craiova, Romania; alexandru.belu94@yahoo.com (A.M.B.); crmilitaru@gmail.com (C.M.); 4 Department of Physiology, University of Medicine and Pharmacy of Craiova, 200349 Craiova, Romania; minapiorescu@gmail.com; 5 Department of Dermatology, Emergency County Hospital, 200642 Craiova, Romania; 6 Cardiomed Hospital, 200032 Craiova, Romania

**Keywords:** lipoprotein(A), myocardical infarction, young people

## Abstract

**Background:** The incidence of acute myocardial infarction (AMI) in young adults has been steadily rising, emphasizing the need for new biomarkers to improve risk stratification. Lipoprotein(a) (Lp(a)), a genetically determined lipoprotein with pro-atherogenic and pro-thrombotic properties, has gained increasing attention in this context. **Methods:** We evaluated serum Lp(a) levels in young patients with AMI and compared them with healthy controls. Associations between elevated Lp(a) levels (≥30 mg/dL) and coronary artery disease patterns were analyzed separately for STEMI and NSTEMI presentations. **Results:** Elevated Lp(a) levels were significantly more common in young patients with AMI compared with healthy controls. Importantly, Lp(a) ≥ 30 mg/dL was strongly associated with multivessel coronary artery disease in NSTEMI, conferring more than a fourfold increased risk. In STEMI, the effect was weaker and largely influenced by concomitant factors such as diabetes and elevated LDL cholesterol. **Conclusions:** These findings highlight key pathophysiological differences between infarct phenotypes and position Lp(a) as a particularly relevant biomarker in young NSTEMI patients. The systematic assessment of Lp(a) may enhance coronary risk stratification and support more tailored secondary prevention strategies.

## 1. Introduction

Over the past decade, the incidence of acute myocardial infarction among young patients has increased steadily, becoming a significant public health issue with major clinical and socio-economic implications [1]. This trend underscores the urgent need for additional biomarkers capable of providing more accurate risk stratification and supporting individualized secondary prevention strategies. Among such biomarkers, lipoprotein(a) [Lp(a)] has attracted particular interest due to its pro-atherogenic and pro-thrombotic properties, as well as its consistent association with premature cardiovascular events [2].

Although myocardial infarction is defined by a common ischemic mechanism, its clinical subtypes—ST-segment elevation myocardial infarction (STEMI) and non-ST-segment elevation myocardial infarction (NSTEMI)—display distinct pathophysiological profiles. STEMI is most often triggered by acute plaque rupture and complete coronary occlusion, whereas NSTEMI is more frequently associated with diffuse, multivessel atherosclerotic disease, reflecting the chronic progression of coronary atherosclerosis [3].

Lipoprotein(a) is considered a major genetically determined risk factor for cardiovascular disease, with circulating levels largely unaffected by environmental factors or lifestyle modifications. Recent studies have consistently demonstrated that elevated Lp(a) concentrations are linked to an increased risk of premature coronary events, independent of LDL cholesterol and other traditional risk factors [4,5].

However, the current body of literature focuses mainly on the role of Lp(a) in the occurrence of myocardial infarction, with far less attention paid to its differential implications in STEMI versus NSTEMI phenotypes and its association with the extent of coronary artery disease in young patients. The novelty of this study lies in the direct comparison of STEMI and NSTEMI phenotypes in young patients and in the differential evaluation of the relationship between Lp(a) and the extent of coronary artery disease. While recent research has linked Lp(a) with the severity of atherosclerotic disease using angiographic scores or with cardiovascular risk in general populations, these studies have not specifically addressed differences between STEMI and NSTEMI in younger individuals. Our results demonstrate that Lp(a) ≥ 30 mg/dL correlates more strongly with multivessel disease in NSTEMI than in STEMI, suggesting incremental values for risk stratification in this phenotype [6,7,8].

## 2. Materials and Methods

### 2.1. Study Design and Population

Our study was conducted on two patient cohorts enrolled over a 6-month period at the Craiova County Emergency Clinical Hospital. We enrolled young patients (<55 years) with acute ST-segment elevation myocardial infarction (STEMI, *n* = 88) and those with acute non-ST-segment elevation myocardial infarction (NSTEMI, *n* = 63) admitted to the Cardiology Clinic. The study was observational. Sample sizes and the compositions of the two groups are documented in the manuscript’s Results section.

Clinical and demographic variables available at admission/assessment were collected: sex, age, body mass index (BMI; analyzed both as a continuous and a categorical variable—normal weight (18.5–24.9 kg/m^2^)/overweight (25.0–29.9 kg/m^2^)/obesity (>30 kg/m^2^), according to World Health Organization criteria, smoking status, and diagnoses of diabetes mellitus and hypertension. The lipid profile included low-density lipoprotein cholesterol (LDL-C) and high-density lipoprotein cholesterol (HDL-C), measured in mg/dL, and lipoprotein(a) (Lp(a)) was quantified; we subsequently set a threshold of 30 mg/dL for stratifying the risk of multivessel coronary involvement (Table 1).

Lipoprotein(a) [Lp(a)] levels were quantified from fasting blood samples using an immunoturbidimetric latex-enhanced assay (Tina-quant^®^ Lipoprotein(a), Roche Diagnostics) on a Cobas Integra 400 Plus analyzer. The assay uses polyclonal antibodies directed against epitopes of apolipoprotein(a). As with all immunochemical methods, Lp(a) measurements may be affected by apo(a) isoform size heterogeneity, potentially leading to under or overestimation of Lp(a) concentrations in individuals with small or large isoforms, respectively. Calibration was performed with manufacturer-supplied reference standards traceable to an international reference material. Analytical validation included assessment of linearity, precision, and accuracy across the 3–120 mg/dL range. Within-run and between-run coefficients of variation were below 5%, in agreement with the manufacturer’s validation data.

### 2.2. Definitions and Measurements

The diagnosis of STEMI/NSTEMI was established according to the center’s standard clinical and blood test, in line with current guidelines by the European Society of Cardiology. The extent of coronary artery disease was assessed by coronary angiography and classified as single-vessel versus multivessel disease. Significant lesions were defined as stenoses greater than 70% of the vessel lumen.

### 2.3. Objectives

The primary objective of the study was to assess and compare lipoprotein(a) [Lp(a)] levels in patients with STEMI and NSTEMI. Secondary objectives included (i) analyses stratified by Lp(a) level (≤30 vs. >30 mg/dL) to compare STEMI with NSTEMI within each stratum, and (ii) evaluation of the association between Lp(a) ≥ 30 mg/dL and the presence of multivessel coronary disease separately in STEMI and in NSTEMI. The subgroup structures (Lp(a) ≤ 30 mg/dL and >30 mg/dL) and the outcome variable (multivessel vs. single-vessel) are detailed in the tables in the Results section.

### 2.4. Statistical Analysis

Continuous variables were summarized as medians and interquartile ranges (IQRs) and categorical variables as counts and percentages. Distributions between the two groups were compared using nonparametric tests for independent samples (Mann–Whitney/Kruskal–Wallis, as appropriate) and χ^2^ or Fisher’s exact tests for categorical variables. To estimate effects on the probability of multivessel disease, separate binary regression models were built for the NSTEMI and STEMI cohorts, reporting relative risks (RRs) with 95% confidence intervals and *p*-values. In adjusted models, predictors included Lp(a) (≥30 vs. <30 mg/dL) and available clinico-metabolic covariates (sex, age, BMI, diabetes mellitus, and LDL-C). Statistical significance was set at *p* < 0.05.

### 2.5. Data Management

Analyses were performed on complete cases using the consolidated database (*n* = 151). Measurement units were kept consistent. The variable structures, BMI categories, and the predefined Lp(a) threshold also derive from the dataset and the tables attached to the manuscript.

## 3. Results

The analysis of clinical and demographic characteristics revealed significant differences between patients with acute myocardial infarction and the control group. In the STEMI cohort, the majority of patients were male (81.8%), a proportion comparable to that observed in NSTEMI patients (76.2%) but significantly higher than in the control group, where the sex distribution was balanced (50% men and 50% women, *p* < 0.001). The median age of STEMI and NSTEMI patients was 48.0 and 50.0 years, respectively, significantly higher than that of the controls, who had a median age of 34.0 years (*p* < 0.001).

Regarding the body mass index, obesity was predominant among STEMI patients (51.1%), while NSTEMI patients were more frequently overweight (66.7%). In contrast, individuals with normal weight predominated in the control group (64.3%), with statistically significant differences between groups (*p* < 0.001). Smoking status showed a high prevalence of smokers in the STEMI group (76.1%) compared with NSTEMI (52.4%) and controls (50.0%, *p* = 0.002).

Diabetes mellitus was present in 27.3% of STEMI patients and 28.6% of NSTEMI patients, whereas none of the control participants had this condition (*p* < 0.001). Arterial hypertension was also frequently encountered among infarction patients, being reported in 64.8% of STEMI and 76.2% of NSTEMI cases compared with its complete absence in the control group (*p* < 0.001).

The lipid profile showed significant differences across groups. LDL-cholesterol values were highest in STEMI patients (median 133 mg/dL) compared with NSTEMI (103 mg/dL) and controls (110 mg/dL, *p* < 0.001). HDL-cholesterol levels were significantly lower in STEMI (39.1 mg/dL) and NSTEMI (41.2 mg/dL) patients compared with controls (55.0 mg/dL, *p* < 0.001).

With regard to lipoprotein(a), a significantly higher proportion of myocardial infarction patients presented values >30 mg/dL, specifically 56.8% in STEMI and 65.1% in NSTEMI, compared with 31.0% in the control group (*p* = 0.002).

**Figure 1:**
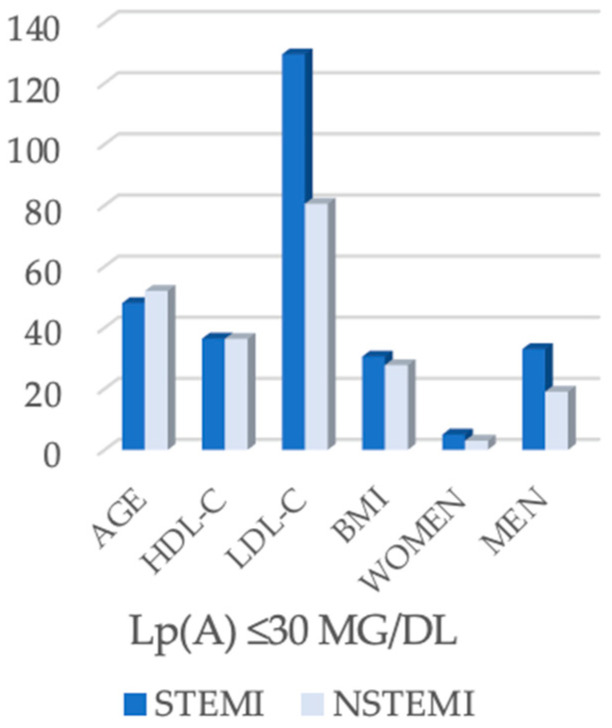
Clinical and biochemical parameters in STEMI and NSTEMI patients with Lp(A) ≤ 30 mg/dL.

The subgroup analysis of patients with low lipoprotein(a) levels (<30 mg/dL, *n* = 60) revealed differences between STEMI and NSTEMI patients (Table 2). The median age was lower in the STEMI cohort (48.0 years) compared with NSTEMI patients (52.0 years, *p* = 0.039). HDL-cholesterol values were similar between the two groups (36.4 mg/dL in STEMI vs. 36.3 mg/dL in NSTEMI, *p* = 0.607), while LDL-cholesterol was significantly higher in STEMI patients (129.4 mg/dL vs. 80.5 mg/dL, *p* < 0.001). BMI was also higher in the STEMI group (30.5 kg/m^2^) compared with NSTEMI (27.7 kg/m^2^, *p* = 0.021). The sex distribution was comparable, with men being more frequently represented in both groups (86.8% STEMI vs. 86.4% NSTEMI, *p* = 1.000) (Figure 1).

With regard to the lipid profile, HDL-cholesterol values did not differ between the two groups (36.4 mg/dL in STEMI vs. 36.3 mg/dL in NSTEMI, *p* = 0.607). In contrast, LDL-cholesterol was significantly higher in STEMI patients (129.4 mg/dL) compared with NSTEMI patients (80.5 mg/dL, *p* < 0.001) (Figure 1).

Body mass index (BMI) was also higher in the STEMI group (30.5 kg/m^2^) compared with NSTEMI (27.7 kg/m^2^, *p* = 0.021), suggesting an association between obesity and infarction type. Sex distribution was similar, with a high proportion of men in both groups (86.8% in STEMI and 86.4% in NSTEMI, *p* = 1.000).

The assessment of coronary artery disease extent showed a comparable prevalence of single-vessel and multivessel lesions between the two subgroups. Single-vessel disease predominated (78.9% in STEMI and 81.8% in NSTEMI), while multivessel involvement was observed in 21.1% of STEMI patients and 18.2% of NSTEMI patients, with no statistically significant differences (*p* = 1.000).

**Figure 2:**
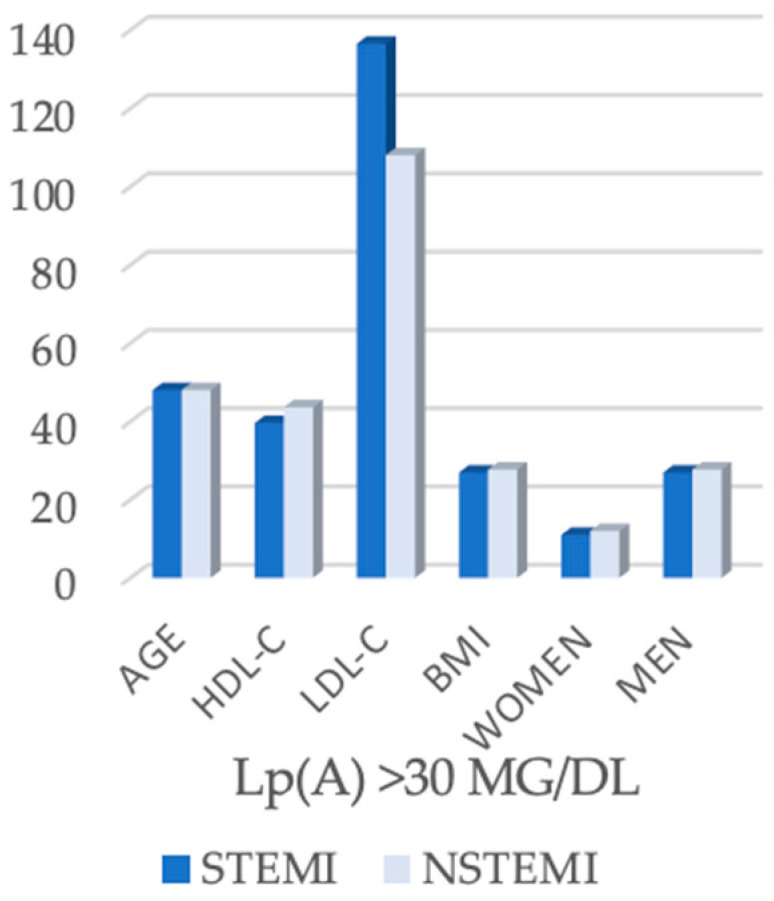
Clinical and biochemical parameters in STEMI and NSTEMI patients with Lp(A) > 30 mg/dL.

In the subgroup of patients with elevated lipoprotein(a) levels (>30 mg/dL, *n* = 91) (Table 3), no significant differences were observed in the median age between STEMI and NSTEMI patients (48.0 years vs. 48.0 years, *p* = 0.251). HDL-cholesterol values were also comparable between the two groups (39.6 mg/dL in STEMI vs. 43.6 mg/dL in NSTEMI, *p* = 0.089) (Figure 2), and the body mass index showed similar values (26.9 vs. 27.7 kg/m^2^, *p* = 0.808). Sex distribution did not differ significantly, although men were more frequently represented in both cohorts (78.0% STEMI vs. 70.7% NSTEMI, *p* = 0.474).

**Figure 3:**
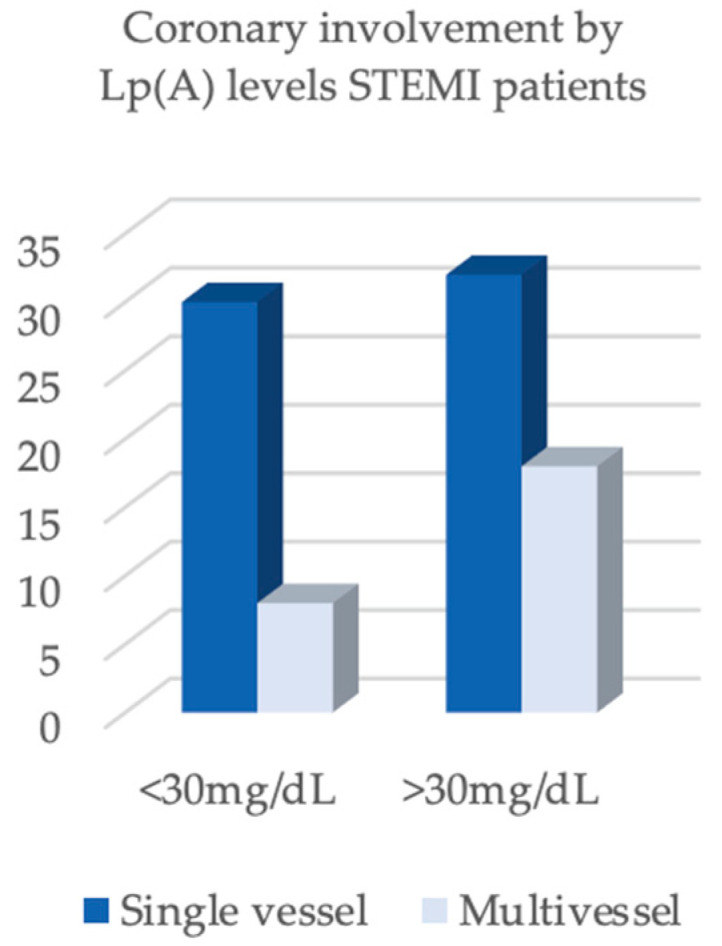
Coronary involvement by Lp(A) levels STEMI patients.

**Figure 4:**
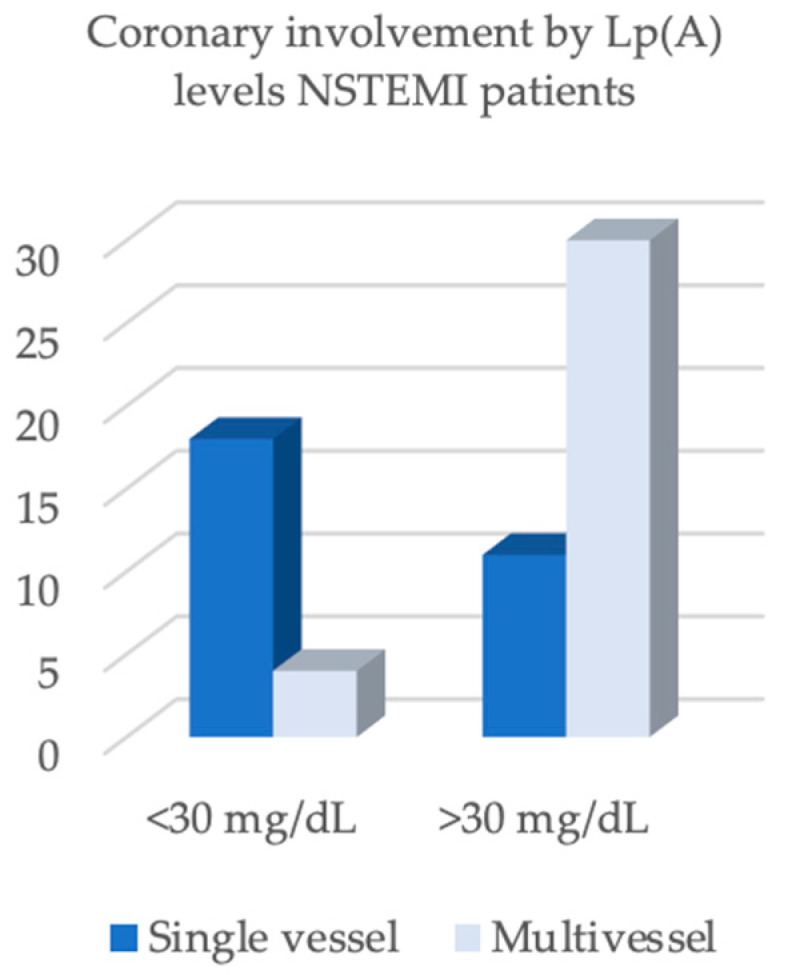
Coronary involvement by Lp(A) levels NSTEMI patients.

In contrast, important differences were found regarding the lipid profile and the extent of coronary artery disease (Figure 3 and Figure 4). LDL-cholesterol was significantly higher in STEMI patients (137.5 mg/dL) compared with NSTEMI patients (108.0 mg/dL, *p* = 0.004). Angiographic analysis revealed a distinct distribution: STEMI patients predominantly presented with single-vessel disease (64.0%), whereas NSTEMI patients more frequently exhibited multivessel disease (73.2%, *p* < 0.001) (Figure 3 and Figure 4).

In summary, while LDL-cholesterol concentrations remained consistently higher among STEMI patients irrespective of lipoprotein(a) levels, elevated Lp(a) was strongly associated with an increased burden of multivessel coronary artery disease, predominantly in the NSTEMI cohort, thereby highlighting its potential contributory role in the pathophysiology and progression of diffuse atherosclerotic disease (Table 2).

In the NSTEMI cohort (*n* = 63, Table 4), elevated lipoprotein(a) [Lp(a) ≥ 30 mg/dL] was strongly associated with multivessel coronary artery disease. The estimated relative risk (RR) was 4.25 (95% CI, 1.73–10.45; *p* = 0.0016), indicating an approximately fourfold higher risk relative to patients with Lp(a) < 30 mg/dL. Among the additional covariates examined, only the body mass index (BMI) demonstrated a statistically significant association: per 5 kg/m^2^ increase, RR 1.55 (95% CI, 1.01–2.38; *p* = 0.043). Male sex (RR 1.54; *p* = 0.130), diabetes (RR 1.29; *p* = 0.285), age (per +5 years; RR 1.05; and *p* = 0.646), and LDL-C (per +10 mg/dL; RR 0.99; and *p* = 0.773) were not statistically significant.

In the STEMI cohort (*n* = 88, Table 5), Lp(a) ≥ 30 mg/dL remained independently associated with multivessel involvement, although with a more modest effect size: adjusted RR 1.78 (95% CI, 1.10–2.90; *p* = 0.020). In this cohort, diabetes was also significantly associated (RR 1.71; 95% CI, and 1.07–2.74; *p* = 0.026), and higher LDL-C conferred additional risk (per +10 mg/dL, RR 1.05; 95% CI, 1.00–1.10; and *p* = 0.044). Male sex (RR 1.37; *p* = 0.329), age (per +5 years; RR 0.98; and *p* = 0.804), and BMI (per +5 kg/m^2^; RR 1.07; and *p* = 0.471) were not significantly associated.

Comparative analyses showed that the association between Lp(a) ≥ 30 mg/dL and multivessel disease was stronger in the NSTEMI than in STEMI cohort (RR 4.25 vs. 1.78) (Table 4 and Table 5). This differential magnitude suggests that elevated Lp(a) is more clinically informative for the extent of coronary involvement in NSTEMI, whereas in STEMI, its effect persists but is accompanied by independent contributions from diabetes and from the atherogenic burden reflected by LDL-C. However, it should be noted that our study was not powered to formally test whether the observed difference in effect size between NSTEMI and STEMI is statistically significant. Therefore, this difference should be interpreted with caution.

Taken together, these findings indicate that an Lp(a) threshold of ≥30 mg/dL is a robust marker of multivessel coronary artery disease, particularly among patients with NSTEMI—whereas in STEMI, Lp(a) remains relevant, but its effect is comparable to that of diabetes and more modest than in NSTEMI, with LDL-C also exerting an independent influence.

## 4. Discussion

Through this study and the results obtained, we sought to evaluate and highlight the role of lipoprotein(a) in young patients with acute myocardial infarction and to compare different cohorts of myocardial infarction presentation, such as STEMI and NSTEMI, by examining its association with the extent of coronary artery disease. Our results show that elevated Lp(a) (≥30 mg/dL) is significantly associated with multivessel disease, with a stronger effect observed in patients with NSTEMI compared to those with STEMI. These findings highlight important pathophysiological differences between the two clinical entities and reinforce the role of Lp(a) as a relevant biomarker in coronary risk stratification [2,9,10].

From a mechanistic perspective, Lp(a) promotes atherothrombosis through multiple pathways: the carriage of oxidized phospholipids, activation of inflammatory cascades (including NLRP3 inflammasome and IL-1β/IL-18), endothelial dysfunction, and smooth muscle cell proliferation, all of which contribute to diffuse atherosclerotic progression. Additionally, the apo(a) moiety interferes with fibrinolysis, leading to persistent thrombosis across multiple vascular territories [11,12]. These mechanisms are more closely aligned with the typical NSTEMI phenotype, characterized by multivessel disease and chronic plaque progression, whereas STEMI usually results from the acute rupture of a single vulnerable plaque, often in the context of metabolic and lifestyle-related risk factors such as high LDL-C and smoking. This mechanistic divergence likely explains the stronger association between elevated Lp(a) and multivessel involvement in NSTEMI observed in our cohort. Similar patterns have been reported in recent imaging and biomarker studies, supporting the role of Lp(a) as a driver of diffuse coronary atherosclerosis [11,12,13,14].

Consistent with data from the literature, we observed that patients with STEMI were generally more obese, more frequently smokers, and had higher LDL-C levels compared with patients with NSTEMI, suggesting a profile of acute plaque rupture triggered by metabolic and lifestyle-related risk factors [15,16,17,18,19]. By contrast, patients with NSTEMI more frequently exhibited multivessel coronary artery disease, reflecting a more diffuse and chronic atherosclerotic burden, within which Lp(a) may exert a stronger pathogenic contribution [20]. This aligns with existing evidence showing that elevated Lp(a) levels promote pro-atherogenic and pro-thrombotic pathways, leading to extensive coronary involvement rather than isolated plaque rupture [21].

Lp(a) concentrations are predominantly (>90%) determined by genetic variability and remain stable throughout life, with minimal influence from environmental or metabolic factors. Therefore, the differences observed between the STEMI and NSTEMI groups in our study are likely related to inherent interindividual genetic variation in Lp(a) levels rather than short-term modifiable risk factors. This concept provides a plausible framework for interpreting the stronger association between elevated Lp(a) and multivessel disease observed in NSTEMI patients.

Regression analyses provide further support for this differential pattern. Among patients with NSTEMI, Lp(a) levels ≥ 30 mg/dL were associated with more than a fourfold increase in the risk of multivessel disease. In contrast, the effect size in STEMI was more modest (RR 1.78), with diabetes and LDL-C also emerging as independent contributors. Taken together, these results indicate that, while Lp(a) is relevant in both clinical settings, it serves as a particularly strong predictor in NSTEMI, where it may help identify patients at a higher risk of diffuse coronary involvement [22].

From a clinical standpoint, our results suggest that systematic Lp(a) testing could enhance risk stratification in young patients with myocardial infarction, particularly those presenting with NSTEMI [23,24]. Early identification of elevated Lp(a) levels may inform more aggressive preventive strategies, including intensive lipid-lowering therapy, emerging Lp(a)-targeted agents currently under development, as well as closer surveillance to optimize secondary prevention [25,26].

Our study also underscores the importance of interpreting Lp(a) levels in the context of other cardiometabolic risk factors. Within the STEMI cohort, diabetes and LDL-C retained significant associations with multivessel disease, underscoring the multifactorial nature of coronary risk in this group. This observation highlights that therapeutic strategies should not only address Lp(a) but should also ensure the rigorous management of traditional risk factors, particularly LDL-C and diabetes, and the early introduction of targeted therapy [27,28,29,30,31].

A key novel aspect of our study is the direct comparison between STEMI and NSTEMI phenotypes in young patients, focusing on the differential association of lipoprotein(a) with the extent of coronary artery disease. While previous studies have examined Lp(a) in relation to the overall cardiovascular risk or severity of coronary atherosclerosis, few have specifically addressed its distinct impact across infarction subtypes in younger populations. By stratifying patients according to Lp(a) levels and analyzing STEMI and NSTEMI separately, our study provides new insights into phenotype-specific pathophysiological patterns and risk stratification.

### Limitations

This study has several limitations. First, the sample size was relatively small and derived from a single center, which may limit the generalizability of the results. Second, the lack of longitudinal follow-up prevents the assessment of the prognostic impact of elevated Lp(a) levels on recurrent events and mortality. Third, our analysis focused on a single biomarker—Lp(a)—without the inclusion of other emerging risk markers such as apoB, hs-CRP, or oxidized phospholipid–apoB. Moreover, Lp(a) concentrations were reported in mg/dL without conversion to nmol/L or adjustment for apo(a) isoforms, which are increasingly recognized as relevant for standardization. Finally, the observational design precludes causal inference. Therefore, our findings should be considered hypothesis-generating, requiring validation in larger, multicenter cohorts with extended follow-up and multimarker panels.

Additionally, the control group was not matched for key baseline characteristics such as age, sex, body mass index, and smoking status. This mismatch may introduce bias and limit the strength of comparisons between patients and controls. Future studies should consider more closely matched control populations to improve the robustness of comparative analyses.

Finally, due to its cross-sectional design, the study does not provide causal answers. The associations identified should be viewed as exploratory and should inform future prospective and pharmacological intervention studies.

## 5. Conclusions

Lipoprotein(a) exerts a substantially greater impact in NSTEMI than in STEMI. Among young patients with elevated Lp(a) levels (≥30 mg/dL), the likelihood of diffuse coronary artery disease is more than fourfold higher in NSTEMI, whereas in STEMI, its contribution is comparatively modest and largely influenced by concomitant risk factors such as diabetes mellitus and elevated LDL cholesterol. These observations underscore fundamental pathophysiological differences between these infarct phenotypes and strongly support the systematic assessment of Lp(a) as an adjunctive tool for coronary risk stratification, particularly in young patients presenting with NSTEMI. Confirmation of these findings through large-scale, multicenter studies with extended follow-up is warranted to further elucidate the prognostic significance of Lp(a) in secondary prevention strategies.

## Figures and Tables

**Table 1:** Clinical, demographic, and laboratory characteristics of the study’s groups.

Parameter		STEMI *n* = 88 No. (%) Median (IQR)	NSTEMI *n* = 63 No. (%) Median (IQR)	CONTROL *n* = 40 No. (%) Median (IQR)	*p*
Gender	Men	72 (81.8%)	48 (76.2%)	20 (50%)	<0.001
	Women	16 (18.2%)	15 (23.8%)	20 (50%)	<0.001
AGE		48.0 (43.8–54.2)	50.0 (46.0–53.0)	34.0 (29.0–40.0)	<0.001
BMI	Normalweight	27 (30.7%)	12 (19.0%)	20 (64.3%)	<0.001
	Overweight	16 (18.2%)	42 (66.7%)	10 (23.8%)	<0.001
	Obesity	45 (51.1%)	9 (14.3%)	10 (23.8%)	<0.001
Smoking status	Smoker	67 (76.1%)	33 (52.4%)	20 (50.0%)	0.002
	Non-smoker	21 (23.9%)	30 (47.6%)	20 (50.0%)	
Diabetes mellitus	YES	24 (27.3%)	18 (28.6%)	0 (0%)	<0.001
	NO	64 (72.7%)	45 (71.4%)	40 (100%)	
HBP status	YES	57 (64.8%)	48 (76.2%)	0 (0%)	<0.001
	NO	31 (35.2%)	15 (23.8%)	40 (100%)	
LDL Cholesterol		133 (100–168)	103 (79.0–121)	110 (90.0–123)	<0.001
HDL Cholesterol		39.1 (32.1–44.8)	41.2 (36.3–51.9)	55.0 (46.4–60.0)	<0.001
Lipoprotein(a) level	Lp(a) ≤ 30 mg/dL	38 (43.2%)	22 (34.9%)	30 (66.67%)	0.002
	Lp(a) > 30 mg/dL	50 (56.8%)	41 (65.1%)	10 (33.33%)	

**Table 2:** Analyses stratified by Lp(a) level ≤ 30 mg/dL.

Parameter	STEMI (*n* = 38)	NSTEMI (*n* = 22)	*p*-Value
Age (years)	48.0 (43.0–54.0)	52.0 (51.0–54.5)	0.039
HDL-C (mg/dL)	36.4 (31.7–44.5)	36.3 (28.5–53.2)	0.607
LDL-C (mg/dL)	129.4 (89.2–157.0)	80.5 (67.9–113.2)	<0.001
BMI (kg/m^2^)	30.5 (26.4–34.4)	27.7 (26.4–28.6)	0.021
Men	33 (86.8%)	19 (86.4%)	1.000
Women	5 (13.2%)	3 (13.6%)	
Single-Vessel Disease	30 (78.9%)	18 (81.8%)	1.000
Multivessel Disease	8 (21.1%)	4 (18.2%)	

**Table 3:** Analyses stratified by Lp(a) level > 30 mg/dL.

Parameter	STEMI (*n* = 50)	NSTEMI (*n* = 41)	*p*-Value
Age (years)	48.0 (45.0–54.8)	48.0 (43.0–50.0)	0.251
HDL-C (mg/dL)	39.6 (33.1–49.9)	43.6 (38.8–44.9)	0.089
LDL-C (mg/dL)	137.5 (108.5–180.6)	108.0 (93.0–145.0)	0.004
BMI (kg/m^2^)	26.9 (24.2–32.1)	27.7 (24.9–29.4)	0.808
Men	39 (78.0%)	29 (70.7%)	0.474
Women	11 (22.0%)	12 (29.3%)	
Single-vessel disease	32 (64.0%)	11 (26.8%)	<0.001
Multivessel disease	18 (36.0%)	30 (73.2%)	

**Table 4:** Logistic regression in NSTEMI cohort.

Predictor	RR	CI	*p*
**Lp(a) ≥ 30 mg/dL (vs. <30)**	**4.25**	**1.73–10.45**	**0.0016**
Gender (men vs. women)	1.54	0.88–2.68	0.130
Diabetes mellitus (yes or no)	1.29	0.81–2.05	0.285
Age (+5 years)	1.05	0.86–1.29	0.646
LDL-C (+10 mg/dL)	0.99	0.95–1.04	0.773
**IMC (+5 kg/m^2^)**	**1.55**	**1.01–2.38**	**0.043**

**Table 5:** Logistic regression in STEMI cohort.

Predictor	RR	CI	*p*
**Lp(a) ≥ 30 mg/dL (vs. <30)**	**1.78**	**1.10–2.90**	**0.020**
Gender (men vs. women)	1.37	0.73–2.56	0.329
**Diabetes mellitus (yes or no)**	**1.71**	**1.07–2.74**	**0.026**
Age (+5 years)	0.98	0.81–1.18	0.804
**LDL-C (+10 mg/dL)**	**1.05**	**1.00–1.10**	**0.044**
IMC (+5 kg/m^2^)	1.07	0.90–1.27	0.471

## Data Availability

The data presented in this study are available on request from the corresponding author due to (Data are not publicly available due to privacy and ethical restrictions).

## Abbreviations

Lp(A)	Lipoprotein(A)
LDL-C	Low density lipoprotein cholesterol
HDL-C	High density lipoprotein cholesterol
HR	Hazard ration
STEMI	ST-elevation myocardial infarction
NSTEMI	Non-ST-elevation myocardial infarction
RR	Relative risk
BMI	Body mass index
CI	Confidence interval
*p*	*p*-value
